# Structure-Based Stabilization of HIV-1 gp120 Enhances Humoral Immune Responses to the Induced Co-Receptor Binding Site

**DOI:** 10.1371/journal.ppat.1000445

**Published:** 2009-05-29

**Authors:** Barna Dey, Krisha Svehla, Ling Xu, Dianne Wycuff, Tongqing Zhou, Gerald Voss, Adhuna Phogat, Bimal K. Chakrabarti, Yuxing Li, George Shaw, Peter D. Kwong, Gary J. Nabel, John R. Mascola, Richard T. Wyatt

**Affiliations:** 1 Vaccine Research Center, National Institute of Allergy and Infectious Diseases, National Institutes of Health, Bethesda, Maryland, United States of America; 2 GlaxoSmithKline Biologicals, Rixensart, Belgium; 3 University of Alabama Birmingham, Birmingham, Alabama, United States of America; University of Pennsylvania School of Medicine, United States of America

## Abstract

The human immunodeficiency virus type 1 (HIV-1) exterior envelope glycoprotein, gp120, possesses conserved binding sites for interaction with the primary virus receptor, CD4, and also for the co-receptor, generally CCR5. Although gp120 is a major target for virus-specific neutralizing antibodies, the gp120 variable elements and its malleable nature contribute to evasion of effective host-neutralizing antibodies. To understand the conformational character and immunogenicity of the gp120 receptor binding sites as potential vaccine targets, we introduced structure-based modifications to stabilize gp120 core proteins (deleted of the gp120 major variable regions) into the conformation recognized by both receptors. Thermodynamic analysis of the re-engineered core with selected ligands revealed significant stabilization of the receptor-binding regions. Stabilization of the co-receptor-binding region was associated with a marked increase in on-rate of ligand binding to this site as determined by surface plasmon resonance. Rabbit immunization studies showed that the conformational stabilization of core proteins, along with increased ligand affinity, was associated with strikingly enhanced humoral immune responses against the co-receptor-binding site. These results demonstrate that structure-based approaches can be exploited to stabilize a conformational site in a large functional protein to enhance immunogenic responses specific for that region.

## Author Summary

Vaccination is an effective means to control worldwide human diseases caused by viruses and other pathogens. Most viral vaccines work by inducing the immune system to generate neutralizing antibodies. The human immunodeficiency virus (HIV) continues to cause huge tolls in terms of human death and disease. The generation of neutralizing antibodies against HIV remains a key but elusive goal for the development of an effective vaccine. Here, we describe a novel approach that uses atomic-level structures of the HIV surface protein, gp120, together with extensive biophysical analysis of this protein, to design modified vaccine candidates. Immunization with these modified gp120 proteins revealed a new relationship between structure-guided protein stability and the efficient elicitation of antibodies against the highly conserved co-receptor binding site of HIV. These data demonstrate the potential for using the design principles established here to develop improved antibody-generating HIV vaccines and for vaccines against other pathogens.

## Introduction

Effective vaccines are an extremely important means to control, and even eradicate (e.g., smallpox) global human pandemics caused by viral and bacterial pathogens (reviewed in [1] and [2]). A major correlate of effective anti-viral vaccines is the elicitation of virus-neutralizing antibodies in vaccinated individuals. With approximately 60 million humans infected with HIV-1 overall, the well-documented global pandemic has resulted in a huge burden of human mortality and morbidity, highlighting the need for an effective vaccine. Structure-based development of HIV-1-specific drugs has been enormously successful, and the application of structure-guided vaccine design is an appealing avenue to advance such efforts (reviewed in [3]). Here, we describe a novel effort to apply structural and thermodynamic analysis to inform the design of vaccine immunogens that induce HIV-1-neutralizing antibodies.

The HIV-1 infection process begins with interaction of the exterior component of the trimeric envelope glycoprotein (Env) complex, gp120, with the primary receptor protein, CD4, present on the host cell surface. Interaction of the Env complex (or functional spike) with CD4, induces exposure of or formation of the co-receptor-binding site on gp120 and enables this glycoprotein to bind chemokine receptor molecules (usually CCR5 or, alternatively, CXCR4) expressed on the surface of a subset of CD4+ lymphocytes (reviewed in [4]). These receptor-induced activation events are followed by fusion of the viral and host cell membranes, mediated by the transmembrane glycoprotein, gp41. It is this series of HIV-1 Env-receptor interactions that are the major focus of research aimed at developing broadly neutralizing antibodies to interrupt the entry process. It is anticipated that if such antibodies can be elicited, they will contribute a major component to protection by an HIV-1 vaccine.

CD4 induces extensive conformational alterations in monomeric gp120 as characterized by unusually large entropic changes following gp120-CD4 interaction and by changes in antigenicity [5]–[9]. The flexible gp120 glycoprotein likely presents multiple conformations to the immune system that are not present on the functional spike [5]. In addition, gp120 possesses conserved antigenic determinants that, in principle, might elicit antibodies capable of neutralizing a broad array of HIV-1 isolates. However, gp120 variable regions and non-neutralizing determinants tend to dominate the elicited immune response [10],[11]. Moreover, extensive Env glycosylation (“glycan shielding”) and conformational masking in the context of the functional spike (i.e., epitope inaccessibility; see [12] and reviewed in [13]) make this glycoprotein a difficult target for broadly neutralizing antibodies [12],[14],[15]. The receptor-binding structures of gp120 are conserved among diverse viral isolates and represent functionally constrained regions that might serve as targets of broadly neutralizing antibodies. However, structural evidence suggests that, within functional spike, the CD4-binding site (CD4bs) is a recessed pocket and the co-receptor-binding site (or CD4-induced region) is either not formed or not exposed until gp120 engages CD4 on target cells [16].

In animal models, passive administration of neutralizing antibodies inhibits HIV-1 infection [17]–[20], demonstrating the proof-of-principle that, if elicited by a vaccine, such antibodies could effectively inhibit viral entry. Typically, effective anti-viral vaccines consist of either live-attenuated or chemically inactivated forms of a given virus. These vaccines usually elicit neutralizing antibodies as a major component of a protective response [2]. However, neither of these approaches has been successful to prevent HIV-1 infection in a safe or effective manner. Much effort has therefore focused on utilizing the HIV-1 envelope glycoproteins as recombinant, subunit vaccines to elicit potent neutralizing antibodies. Due to the aforementioned Env variability, here we focused on eliciting antibodies against the functionally and structurally conserved receptor-binding regions of gp120.

Several studies attempted to elucidate the biophysical factors of the antigen that effect the maturation of host antibody responses [21],[22]. However, no study to date has tested the impact of conformational stabilization and increased ligand affinity on enhancing the immune responses against discrete conformational regions in the context of a large functional protein. Here, we test the concept that conformational fixation of the conserved receptor-binding sites on the surface of gp120 would enhance elicitation of antibody responses against those target sites. The approach is based upon the high-resolution crystal structures of core gp120 protein in a ternary complex with CD4 and the co-receptor mimetic, CD4-induced (CD4i) antibody, 17b, and the unliganded SIV core structure [16],[23]. The structural information, consistent with the thermodynamic analysis, indicates that major structural rearrangements occur within gp120 following interaction with CD4 [23]. Previously, we exploited this information to design core gp120 molecules with up to 50% stabilization of the CD4bs, and one such protein, Ds12F123, was co-crystallized with the broadly neutralizing anti-HIV-1 antibody, b12 [24]. However, limitations in protein expression prevented us from introducing additional stabilizing mutations into this molecule to achieve greater conformational stabilization.

In the present study, we re-designed the core gp120, based upon new available structures [25], to enhance protein folding and expression, and layered upon this, additional mutations to stabilize the CD4-binding site as well as the co-receptor-binding region. Detailed conformational characterization of the receptor-binding sites of these modified proteins are presented. We tested the effects of the stabilizing mutations in regards to the elicitation of antibodies in small animals. We demonstrate that the novel mutagenic stabilization of a discontinuous epitope, typified by an increased on-rate of ligand binding to this region, dramatically increased the immunogenicity and neutralizing capacity of elicited antibodies specific for that epitope region.

## Results

### gp120 Core Redesign Permits the Introduction of Four Stabilizing Cysteine Pairs

**Figure 1:**
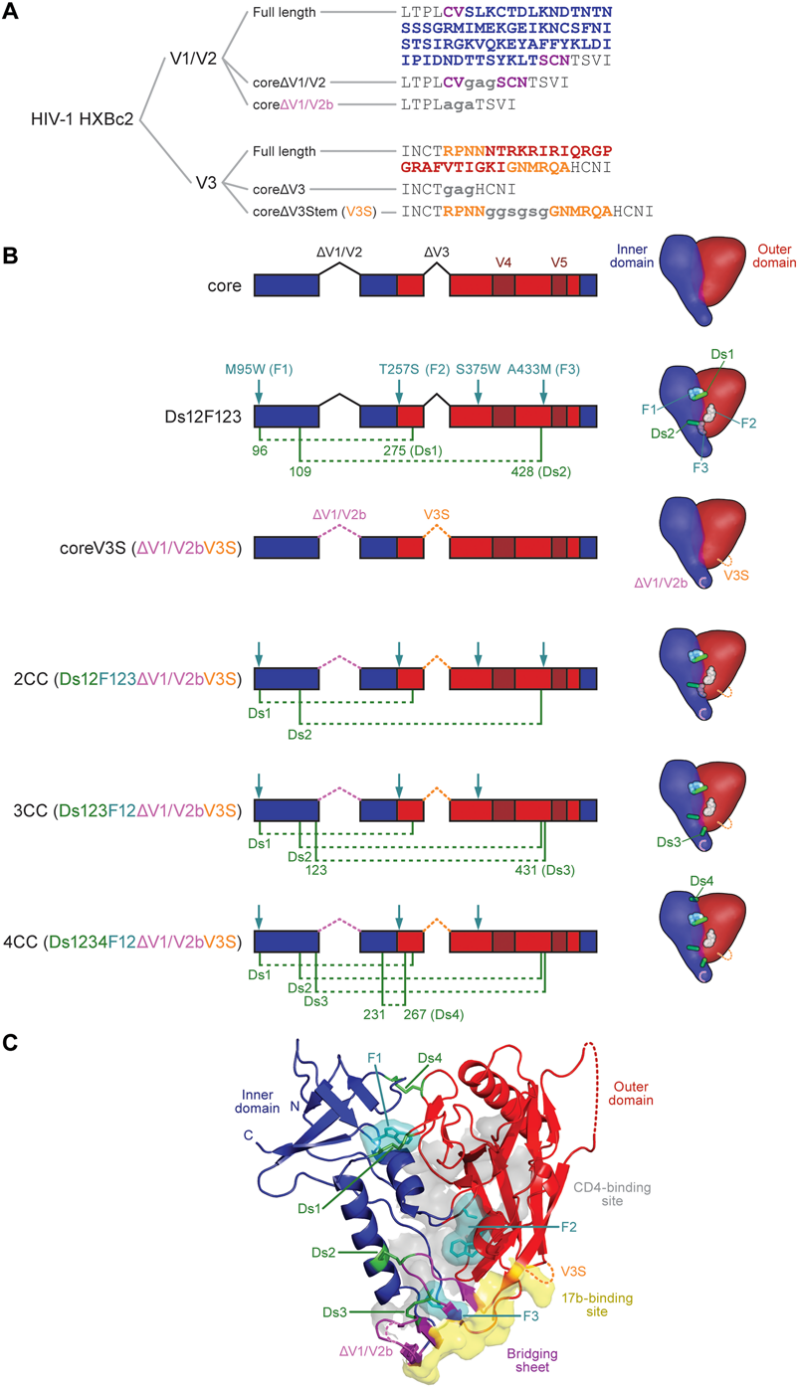
Structural elements of the HXBc2 gp120 core protein and its stabilized derivatives. A. Amino acid sequences of the V1/V2 loop and the V3 loop as present in full-length gp120, in the previously crystallized core protein and in coreV3S protein. Linker sequences are in lower case. B. Linear diagram of core and coreV3S derivatives showing positions of V1/V2- and V3 loop deletions, cavity-filling mutations (F, teal arrows) and paired disulfide mutations (Ds, green dotted lines). The schematics depict surface models of each corresponding core derivative indicating relative positions of the mutations within inner domain (blue) and outer domain (red). C. Ribbon diagram of HXBc2 core gp120 crystal structure with all stabilizing mutations modeled on it. Indicated are the modifications of V1V2- and V3 loops (labeled dashed lines), the CD4 binding surface (translucent gray), the 17b epitope surface contacts (yellow) and the beta strands comprising the bridging sheet sub-domain (purple). Note the proximity of Ds2 and Ds3 to both the CD4 binding site and the bridging sheet. 17b is the prototypic co-receptor-binding-site-directed antibody, which inhibits gp120-CD4 interaction with co-receptor, and serves as surrogate for co-receptor N-terminal interaction with the gp120 core. Conserved V3 loop elements (not shown) also contribute to gp120-CD4-co-receptor interaction.

To focus the immune response onto the conserved receptor-binding sites, it is important to remove immunodominant regions, such as the V1/V2 and V3 hypervariable loops. Previously, loop truncations demonstrated that such removal was possible; however, structural analysis suggested more optimal designs were feasible [16],[25],[26]. For example, the structure of core gp120 with intact V3 loop showed that the previously published Gly-Ala-Gly substitution of V3 residues 298–329 (to accomplish deletion of V3) removed four hydrogen-bonds from β-strand 12 and five hydrogen-bonds from β-strand 13 [25]. We modeled a new substitution (V3S) that retained these hydrogen bonds, and added a longer linker (Figure 1A). Further structural analysis indicated that additional trimming of the flexible V1V2 loop to eliminate a naturally occurring cysteine pair might facilitate accommodation of additional pairs of stabilizing cysteines elsewhere in the molecule. Accordingly, a more minimal loop (V1/V2b) was modeled with a type II turn connecting strands β2 and β3, replacing nine residues (CVGAGSCNT) with an Ala-Gly-Ala tri-peptide (see Figure 1).

To reduce conformational flexibility and lock core gp120 into its receptor-bound state, we used two tactics: filling hydrophobic pockets and adding inter-domain disulfides. We previously described cavity-filling or “F mutations” to fill the Phe-43 pocket (where critical contacts are made for CD4 binding) and other gp120 cavities [24],[27],[28], and also described the introduction of inter-domain cysteine pairs (disulfides or Ds mutations) [24]. Here, we used a combination of loop alterations, F mutations and Ds mutations, to create four new immunogens. The “coreV3S” contained the V1/V2b and V3S alterations. Meanwhile “2CC”, “3CC” and “4CC” involved 2, 3 and 4 additional inter-domain disulfides, in concert with the gp120 cavity-filling mutations (F1, F2 and/or F3; Figure 1B and 1C). We also expressed two previously described immunogens, core and Ds12F123 [24], as controls. The new designs resulted in the expression of well-folded gp120 proteins as assessed by their interactions with conformational ligands 17b and b12 (see below). Protein purity and molecular mass were determined by SDS-PAGE analysis followed by Coomassie blue staining (Figure S1).

### Variable Loop Structural Elements Influence Formation of the Receptor-Binding Sites

**Figure 2:**
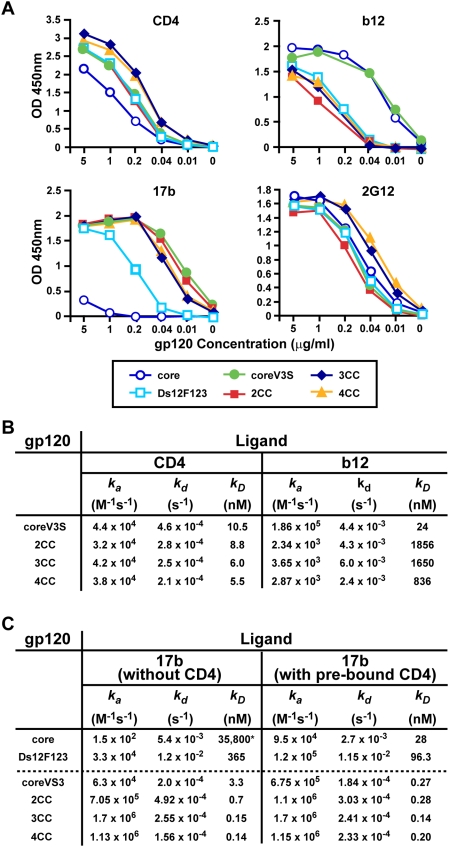
Antigenic analysis of unmodified and structurally stabilized core envelope variants by ELISA and SPR. A. ELISA plates were coated with ligand (2 µg/ml), reacted with 5-fold serial dilutions of affinity-purified envelope glycoproteins (starting at 5 µg/ml) and detected with 1∶2500 dilution of rabbit immune sera raised against HXBc2 core protein (unmodified). Upper left, binding to soluble human CD4 (4-domain; Progenics); upper right, binding to b12; lower left, binding to 17b; lower right, binding to 2G12. Margins of error from duplicate wells were negligible in all cases. B and C. Binding rate constants for interactions of stabilized gp120 core proteins with sCD4, b12 and 17b. Approximately 500 RU each of 17b, sCD4 and b12 were coated on CM5 chip. Two-fold serial dilutions of each gp120 protein were allowed to bind to the surfaces for 3 min followed by dissociation for 5 min. The kinetic constants were obtained by fitting the curves to 1∶1 Langmuir binding model. B. Kinetics of CD4 and b12 interactions. C. Kinetics of 17b binding to gp120 variants without or with pre-exposure to 10-fold molar excess of sCD4. *Data obtained from very low binding interaction (maximum RU of 8.5).

The purified proteins were tested for recognition by ligands directed against the receptor binding sites, first by ELISA (Figure 2A). In agreement with our previous data, the stabilizing mutations in Ds12F123 enhanced affinity for CD4 binding over that of the parent core protein. Interestingly, the coreV3S protein displayed slightly increased CD4 affinity even without any additional stabilizing modifications, suggesting that the V1V2b and V3S modifications influence formation, stability or accessibility of the CD4 binding region on the gp120 core. Addition of stabilizing mutations to coreV3S protein, however, did not increase the already high affinity binding of CD4 to the modified proteins. We also assessed recognition of the proteins by b12, a CD4bs-directed antibody that recognizes a surface similar to but not identical with that of CD4 [24]. The core and the coreV3S proteins both displayed similar recognition by b12, suggesting that the modified truncations of the V1V2- and the V3 loop did not impact upon b12 binding. However, addition of the stabilizing mutations affected b12 binding to different degrees irrespective of the core context (see Figure 2). These findings are consistent with our earlier observation that the same set of stabilizing mutations reduce b12 affinity to some degree [24]. However, the greater impact of the stabilizing mutations on b12 affinity observed in this study may be a result of the coreV3S protein context or, for the ELISA, by the detection reagent used (i.e., rabbit sera raised against the unmodified core gp120 protein).

We then tested effects on the gp120 co-receptor-binding site assessed by recognition of the cores by the co-receptor mimetic antibody, 17b [6]. As expected, the original core gp120 protein was not recognized by 17b, in contrast to the partially stabilized Ds12F123 protein (which possesses 2 pairs of cysteines; see also [24]). However, the V3S modifications facilitated increased recognition by 17b (closed circles) even in the absence of CD4 or stabilizing mutations. Incorporation of 2, 3 or 4 cysteine pairs did not alter the avid recognition of the coreV3S protein by 17b as determined by ELISA (Figure 2A). These results indicated that somewhat unexpectedly, the V3S structural elements play a critical role in the formation of the 17b-associated co-receptor-binding site.

We also determined effects of the structural alterations on the recognition of gp120 by ligands that bound outside the CD4 and co-receptor regions. We selected the monoclonal antibody 2G12, which binds to a conformational glycan epitope on the outer domain of gp120 that is distal from the CD4bs. The 2CC protein displayed slightly reduced affinity whereas the 3CC and 4CC proteins showed somewhat increased affinity for 2G12. These small but unanticipated differences in affinities were not contributed by differences in the amounts of proteins used since equal protein quantities were confirmed by both optical density and SDS gel analysis (data not shown).

### Stabilizing Mutations Affect Ligand Affinity to the Co-Receptor Site by Increased On-Rate

Since the putative stabilizing mutations influenced recognition by the receptor-site-directed ligands, we performed SPR studies to identify the contributions of the individual rate constants in regards to the changes in affinity for these ligands. As shown in Figure 2B, the on-rates (*k_a_*) of CD4 binding to the stabilized cores remained nearly unchanged relative to the unmodified core. However, there was a subtle and gradual decrease in off-rates upon addition of the cysteine pairs, leading up to twofold increase in CD4 affinity (10.5 nM for coreV3S versus 5.5 nM for 4CC). For b12 binding, the on-rates were significantly reduced for the stabilized proteins as reflected in the overall affinities. For the kinetic analysis of 17b binding (Figure 2C), we included the core protein (without the V3S modifications) to determine the influence of V3S modification on the antigenicity of the co-receptor-binding site. The core protein was recognized by 17b with extremely low affinity, which was not detectable in the ELISA format (see also Figure 2A). In stark contrast, the newly designed coreV3S protein is recognized by 17b with remarkably high affinity (3 nM), even in the absence of CD4. Addition of the stabilizing mutations did not alter the off-rates of 17b interaction to any of the coreV3S variants. However, the on-rates increased significantly, ranging from increases of 11- (for 2CC) to 27- (for 3CC) to 18-fold (for 4CC) over the on-rate observed for 17b binding to coreV3S. Therefore, the enhancement of 17b affinity for the series of V3S-stabilized cores was directly correlated with an increase in the on-rate of antibody binding.

To investigate the influences of CD4 interaction with gp120 on 17b binding in the coreV3S protein context, the V3S protein variants were pre-incubated with 10-fold molar excess of sCD4 and the protein mixtures were then analyzed by SPR. As expected, the previously crystallized core protein showed high affinity (28 nM) binding to 17b in the presence of CD4, with nearly undetectable affinity in the absence of CD4. Note, however, that the coreV3S displayed nanomolar affinity even in the absence of CD4, implicating greatly the V3S modifications on stabilization of the 17b epitope, and perhaps the bridging sheet itself (see Figure 1 and [16]). The association rate constant for 17b binding to coreV3S was increased 10-fold in the presence of CD4, resulting in nearly a 10-fold increase in the observed affinity. The 2CC protein exhibited only a 1.5-fold gain in the on-rate of 17b binding and a 2.5-fold increase in overall 17b affinity in the presence of CD4. For cores containing the 3CC or 4CC mutations, pre-incubation with CD4 did not alter 17b affinity, suggesting that the 3CC and 4CC mutations mimic closely the conformational effects induced by CD4 relative to formation or stabilization of the co-receptor-binding site.

### Thermodynamic Analysis Confirms Mutagenic Stabilization of the CD4–Binding Site and the Co-Receptor Binding Region

**Figure 3:**
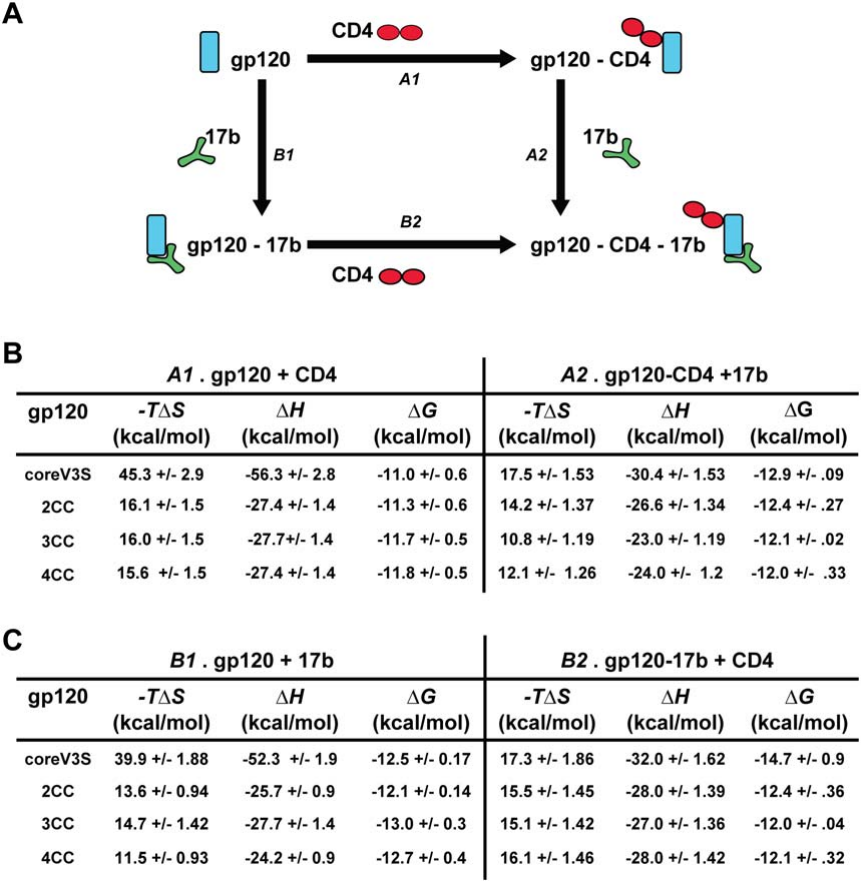
Thermodynamic values of sCD4 and 17b interactions with gp120 variants measured by ITC at 37°C. A. Schematic of complete thermodynamic cycles showing two possible orders of reactions. B and C. Changes in enthalpy (*ΔH*), entropy (*−TΔS*) and free energy (*ΔG*) upon ligand interactions with gp120. B. Binding to CD4 (*A1*) followed by binding to 17b (*A2*). C. Binding of 17b (*B1*) followed by binding to CD4 (*B2*).

To evaluate the extent of stabilization of the receptor-binding sites on gp120, we performed isothermal titration calorimetry (ITC) and determined the apparent change in enthalpy (*ΔH*) and entropy (*−TΔS*) upon binding of coreV3S variants to the primary receptor, CD4, or to the co-receptor mimetic antibody, 17b. The complete thermodynamic cycle of CD4 and 17b binding were measured in the two possible orders: *A* and *B* (Figure 3A). In order *A*, CD4 was combined with gp120 (*A1*), followed by 17b binding to the gp120-CD4 complex (*A2*); in order *B*, 17b was combined with gp120 (*B1*), followed by CD4 binding to the gp120-17b complex (*B2*). The thermodynamic values of these interactions are summarized in Figure 3B and 3C. In *A1* reactions, the enthalpy of CD4 interaction was approximately 56 kcal/mol for the coreV3S protein and approximately 27 kcal/mol for each of the mutants, indicating that approximately 50% less bond formations/reformations and/or solvent displacement occurred when the stabilizing mutations were present. This observation was supported by approximately 65% reduction in change in entropy for the mutants (approximately 16 kcal/mol) compared to the coreV3S protein (45 kcal/mol), indicating that the mutations substantially stabilized the coreV3S protein into the CD4-bound conformation. Interestingly, the calculated entropy values were similar for all the mutants, suggesting that Ds12F123 mutations, present in 2CC, account for most of the effect in stabilizing the CD4-binding region. In *A2* reaction, addition of 17b to the coreV3S-CD4 complexes introduced further conformational rigidity in gp120, as accounted by an entropy change of 17.5 kcal/mol. However, entropy of this interaction was significantly decreased by the stabilizing mutations in 2CC and 3CC, indicating that besides stabilizing the CD4-binding site, the structure-guided mutations further stabilized the co-receptor-binding site in a manner beyond that achievable by interaction with CD4 itself.

To measure the effects of the mutations on stabilizing the co-receptor-binding site, the 17b antibody was titrated with each envelope variant (*B1* reactions). 17b binding to the coreV3S yielded approximately 52 kcal/mol of favorable enthalpy and approximately 40 kcal/mol of change in compensating unfavorable entropy, suggesting that 17b binding alone can induce similarly large conformational (and/or solvation) effects to the gp120 core. The stabilizing mutations reduced the enthalpy change during the *B1* reaction by almost 50%. Consistent with these data, we observed as well a significant decrease in the entropy change in the presence of 2, 3 or 4 pairs of cysteines, leading up to 75% reduced entropy for 17b interaction as compared to the ∼40 kcal/mol value obtained for 17b-coreV3S. These results indicated that the selected mutations significantly stabilized the conformation of the co-receptor-binding site. We then measured the thermodynamics of CD4 binding to these gp120-17b complexes (*B2* reactions). CD4 binding to coreV3S-17b complexes resulted in a 17.3 kcal/mol of entropy change. However, the entropy value of CD4 binding to 17b complexes with 2CC, 3CC or 4CC proteins, each containing the respective stabilizing mutations, did not change relative to the parental protein. The entropy of CD4 binding to gp120-17b complexes (approximately 16 kcal/mol) therefore perhaps resulted from reduced flexibility of gp120 elements distal from the CD4-binding pocket, and/or from solvent effects. The *B2* reactions also showed less negative *ΔG* values in the presence of the stabilizing mutations, suggesting that 17b alone can induce a most thermodynamically favorable conformation of the CD4-binding site, and presence of the current set of stabilizing mutations actually conferred a slightly negative impact on the 17b-induced CD4-binding region.

### Stabilization Selectively Enhances Immunogenicity of the Co-Receptor Binding Region

The kinetic and thermodynamic characterizations of the coreV3S envelope variants revealed considerable stabilization of both the CD4bs and the co-receptor binding site, associated particularly with enhanced on-rate and affinity by 17b. Therefore, we tested impact of these modifications on elicitation of antibody responses *in vivo*. To allow better statistical analysis, we immunized 14 rabbits with each protein immunogen. The overall immune response after each inoculation was analyzed by ELISA to measure IgG binding to either core or to coreV3S proteins. In all cases (except for BSA), high titers of anti-gp120 antibodies were detected after two inoculations, with end-point titers reaching 1.5×10^5^ (Figure S2). The binding titers did not substantially increase with additional inoculations.

**Figure 4:**
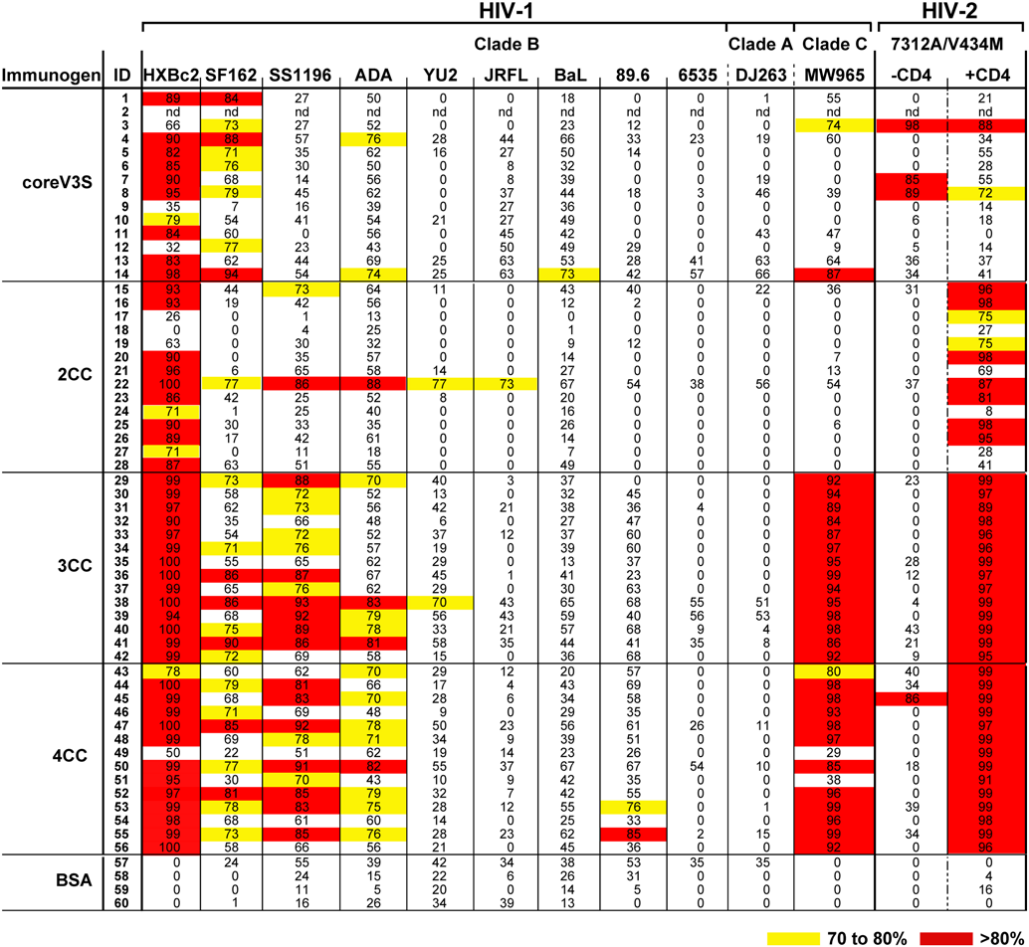
Neutralization profile of fivefold diluted rabbit immune sera tested against a panel of HIV-1 and HIV-2 isolates. All sera tested were collected after four inoculations. Neutralization by preimmune sera was used as negative control for serum reactivity.

After four inoculations, we sought to analyze the overall breadth of HIV neutralization elicited by these immunogens. Due to the large number of sera, we screened the neutralization activity at a 1∶5 dilution of each serum against viruses pseudotyped with clade B (9 isolates) or clade C (1 isolate) or clade A (1 isolate) HIV-1 envelope glycoproteins. The results, shown as percent neutralization of viral entry, are summarized in Figure 4. Autologous (HXBc2) neutralization was achieved by all sera. Interestingly, although the breadth was somewhat limited, the 3CC and 4CC stabilized V3S immunogens elicited a trend of higher neutralization responses against several primary HIV-1 clade B isolates, namely SF162, SS1196 and ADA (a typically neutralization-resistant isolate), and a clade C isolate, MW965 when compared to responses elicited by the coreV3S (Figure 4). In addition, we analyzed core and Ds12F123 proteins (lacking the V3S modifications) for immunogenicity and obtained similar results to the V3S equivalents (Figure S3). Because percent neutralization is a rough approximation of the actual inhibitory titer, we confirmed these data by deriving inhibitory dilution 50% values (ID_50_) of all sera for selected isolates (HXBc2, SS1196 and MW965; see Figure S4).

**Figure 5:**
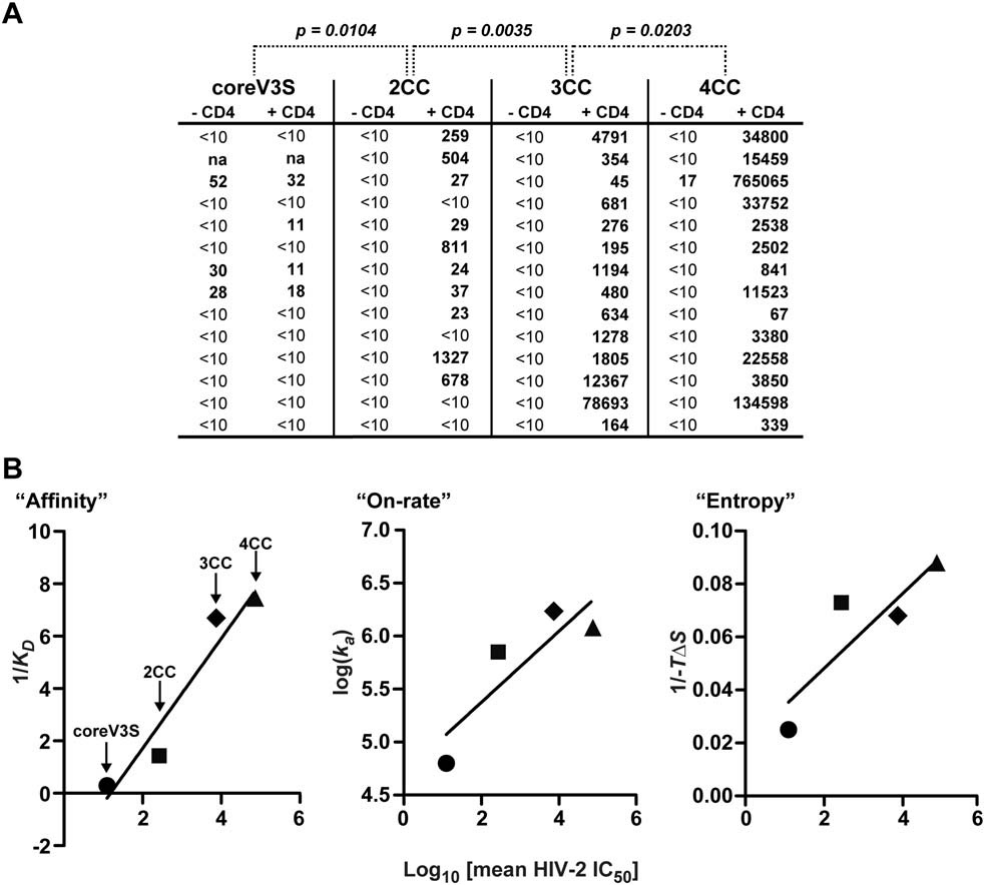
Statistical correlation of HIV-2 neutralization titers with kinetic and thermodynamic properties. A. ID_50_ values of HIV-2 neutralization by rabbit immune sera, performed in the absence and presence of sCD4. The statistical significance (*p* value) of the increase in HIV-2-neutralization titer (in the presence of sCD4) with the increase in the number of stabilizing cysteines (2CC, 3CC, 4CC) was analyzed by Mann-Whitney test. B. Linear regression analysis showing correlations of HIV-2-neutralization titer with kinetic and thermodynamic parameters. Left, with reciprocal of affinity (1/k_D_); middle, with reciprocal of the association rate constant (1/k_a_); right, with the percent of epitope stabilization, as measured from the entropy change (−*TΔS*) relative to that of coreV3S.

Since the cysteine-based mutagenesis resulted in significant stabilization and enhanced ligand affinity of the co-receptor-binding site, we employed an assay which detects the presence of functionally active antibodies specific for the co-receptor binding site that is conserved between HIV-1 and HIV-2 (see Figure 5A and Methods; [29]). Most sera elicited by coreV3S demonstrated little cross neutralization of the HIV-2 isolate (see Figure 5A). However, in stark contrast, very potent HIV-2 neutralization responses were elicited by the stabilized core immunogens, 3CC and 4CC. Moderate neutralization was elicited by the 2CC immunogen. In the 3CC and 4CC elicited sera, we observed low-titer and inconsistent neutralization of SF162, SS1196 and ADA that parallels the more consistent neutralization of a particular clade C HIV-1 isolate, MW965, and the HIV-2 isolate, 7312AV434M, by these sera. Interestingly, both of the latter two isolates are known to be sensitive to co-receptor binding site-directed antibodies under the conditions tested.

Following the initial neutralization analysis at a single dilution of the sera, we determined ID_50_ values of all immune sera against the indicator HIV-2 isolate (Figure 5A). Compared to the non-stabilized coreV3S, the stabilized proteins elicited significantly more potent HIV-2 neutralizing responses in the order of 4CC>3CC>2CC>coreV3S. The linear regression analysis of the HIV-2 neutralization titers and corresponding immunogen properties (ligand affinity, on-rate of ligand binding and stabilization of the 17b epitope) showed distinct linear correlations in all cases (Figure 5B).

In a parallel immunogenicity study, performed in guinea pigs, the V3S or 3CC modifications were introduced into a DNA prime, recombinant adenovirus (rAd) regimen in a gp120 core context or in the previously described gp145ΔCFI and gp140ΔCFI contexts (Figure S5, panel A; [30]). High serum titers of HIV-2 cross-neutralizing antibodies were detected in guinea pigs that were inoculated with the 3CC-containing DNA/rAd immunogens only (Figure S5, panel B).

Next, we examined the effects of conformational stabilization on the elicitation of antibodies to the CD4 binding region. Since there is no HIV neutralization assay available yet to specifically map serum immune responses against the CD4bs, we performed competition ELISA experiments with CD4 as previously described (Figure S6; [28]). We also established a similar competition assay with the CD4bs antibody, b12, based upon our observation that the presence of excess 17b antibody does not affect the binding of b12 to the coreV3S protein (Figure S7). Results from both competition assays indicated that all three of the stabilized immunogens elicited CD4bs-directed antibodies, although, in particular instances, to lesser extents compared to the coreV3S immunogen. Therefore, the modest neutralization capacity elicited by the coreV3S variants (Figure 4) indicated that if we have elicited neutralizing antibodies against the CD4 binding region, they are not of the breadth or potency of b12 or CD4 itself. These data were consistent with binding analysis following the differential adsorptions on selected sera described below.

### Stabilization Shifts the Elicited Antibody Binding and Neutralization Specificity

**Figure 6:**
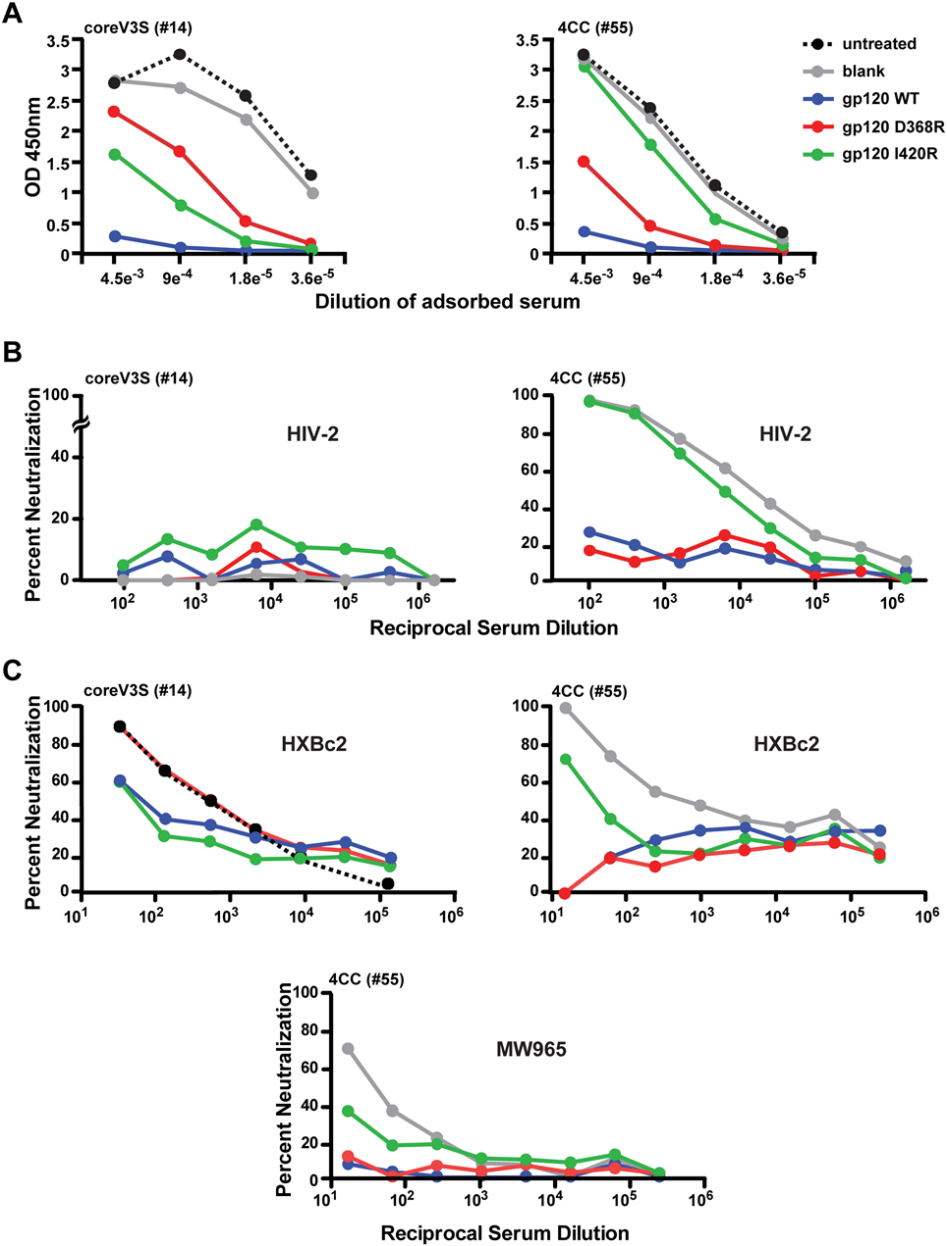
Percent neutralization of pseudotyped HIV isolates by differentially adsorbed flow-through fractions of rabbit immune sera. A. ELISA experiments to determine gp120-binding titers of serum fractions following selective adsorptions. Immune sera from the unmodified (coreV3S) and the most-stabilized (4CC) immunogen groups were incubated with uncoated dynal beads (blank) or dynal beads coated with BSA or gp120WT or gp120-D368R or gp120-I420R proteins. Starting at 11-fold dilution, fivefold serial dilutions of the FTs, from these reactions were tested for binding to gp120 on ELISA plates. Untreated serum or serum FTs from reactions with blank beads and were used as positive controls and FTs after incubation with gp120 beads were used as negative controls for binding. B. Neutralization of HIV-2 7312/V434M isolate by immune sera FTs following differential adsorptions. Neutralization was performed in the presence of 0.5 µg/ml of soluble CD4 (sCD4). C. Neutralization of the HIV-1 clade B isolate, HXBc2 (top panels) and the clade C isolate, MW965 (bottom panel). Margins of error obtained from duplicate reactions were negligible.

The HIV-1 HXBc2 gp120 variants coreV3S and 4CC proteins, elicited strikingly different levels of CD4i antibodies as determined by the HIV-2 cross-neutralization assay (Figure 5). However, sera derived from immunized animals from both the coreV3S and the 4CC group potently neutralized the autologous virus, HXBc2. We therefore sought to characterize the target specificity of the elicited neutralizing antibodies. We performed differential adsorption of antibody subpopulations from each serum in a previously described process [31],[32], followed by binding analysis and neutralization assays as described below. Due to the necessity to adsorb out all binding antibodies in this process by gp120 protein excess, this is not a high throughput assay in hyper-immune animals. Therefore, immune sera from one coreV3S-immunized rabbit and one 4CC-immunized rabbit were selected for the analysis. The sera were incubated with Dynabeads covalently conjugated with one of the following indicator proteins: gp120WT, to adsorb out all gp120-directed antibodies; gp120D368R, to adsorb out all but CD4bs-directed antibodies, and gp120I420R, to adsorb out all but CD4i antibodies. Following selective adsorptions, performed in gp120 excess, the flow-throughs from these reactions were first analyzed by ELISA to verify completion of adsorption and to determine relative prevalence of each antibody type (Figure 6A). Complete adsorption in each reaction was confirmed by the lack of binding of the adsorption flow-throughs (FT; containing non-adsorbed antibodies) to the same protein target that was attached to the corresponding beads. The titers of either CD4bs-directed antibodies or co-receptor-binding site-directed antibodies were determined from binding of corresponding depleted serum to gp120WT protein. The coreV3S protein elicited much higher titer of CD4bs-directed antibodies than CD4i antibodies (Figure 6A, left panel), and the conformationally stabilized 4CC protein dramatically shifted this response towards eliciting much higher CD4i antibodies than CD4bs-directed antibodies (right panel). This type of differential analysis (previously described in reference [32]) is subject to less off-target effects than are cross-competition assays and is a more definitive means to map binding specificities delineated by selected gp120 point mutations.

Next, the potency of the selectively adsorbed immune sera fractions was tested in selected neutralization assays. The coreV3S protein elicited low levels of CD4i binding site antibodies (Figure 6A, left panel), but the serum did not neutralize HIV-2 (Figure 6B, left panel). In contrast, only the CD4i antibody population (gp120 I420R FT) of the 4CC-immunized sera potently neutralized the HIV-2 isolate (Figure 6B, right panel) and, as well, the highly 17b-sensitive clade C isolate, MW965 (Figure 6C, bottom panel). Similarly, only the CD4i antibody population in the 4CC serum neutralized yet another HIV-1 clade B isolate, SS1196, but the potency of neutralization observed under the experimental conditions used here was relatively low (data not shown).

The same adsorbed immune serum fractions were then analyzed in an HXBc2 neutralization assay. This sensitive isolate is neutralized by both non-potent CD4bs-directed antibodies and by the non-potent co-receptor-binding-site-directed antibodies. As shown in Figure 6C, left panel, coreV3S immune serum mediated neutralization of HXBc2 mostly by CD4bs-directed antibodies (gp120D368R FT), the first time elicitation of antibodies of such specificity by an Env-based immunogen has been demonstrated. Analysis of serum ID_50_ values (Figure S3) indicated that the 3CC protein trended toward the elicitation of slightly higher HXBc2 neutralization titers than those elicited by the 4CC protein while the potency of HIV-2 neutralization was reversed between these groups. The data are another indication that the stabilizing mutations affect the neutralization specificity elicited by the core proteins.

## Discussion

Due to the pressing need for an effective HIV-1 vaccine, and due to the limits of current Env-based immunogens to elicit neutralization breadth, we pursued HIV-1 Env structure-guided immunogen design to determine if this line of investigation will better elicit virus neutralizing antibodies. Here, we demonstrate that the structure-based redesigning of the HIV-1 envelope core glycoprotein increased folding and expression of a series of related, mutagenically stabilized molecules. Structure-guided protein design led to stabilization of both the CD4-binding site and the co-receptor-binding region of gp120. The data clearly demonstrated that thermodynamic stabilization of the co-receptor-binding site was associated with a marked increase in the on-rate of binding of the co-receptor mimetic, 17b antibody. Furthermore, when immunized into small animals, stabilization of the gp120 core resulted in a dramatic enhancement of the functional antibody response against the CD4-enhanced co-receptor binding region shared by HIV-1 and HIV-2. These results suggest that, in general, specific regions of an immunogen might be rendered more immunodominant by direct conformational stabilization (in this case, cysteine-pair mutagenesis) of that region resulting in reduced entropy. These results are appealing from a thermodynamic perspective, as the ligand affinity (*ΔG*) can be more favorable with a reduction in entropy (−*TΔS*). This thermodynamic relationship would predict that if a given epitope (or circumscribed region) is pre-fixed into a desired conformation, a ligand (i.e.,17b or perhaps a “naïve” B cell receptor) specific for that site will not be required to initiate “induced-fit” [33] and will therefore bind to the site with an increased on-rate [34]. Potentially, the overall affinity may increase (assuming no negative impact on the off-rate), and in regards to the B cell receptor, a faster on-rate may enhance epitope recognition, resulting in more efficient activation of that B cell. The finding described in this study, demonstrate that conformational stabilization of a discrete protein region can alter the quantity and quality of the antibody response to that protein region. They suggest that ligand stabilization or improved ligand affinity, especially ligand on-rate, might be used as parameters to focus functional antibody responses on specific regions on the surface of a complex, conformationally sensitive and multi-epitope protein.

Recently we reported that the elicitation of co-receptor-directed antibodies is dependent upon interaction of gp140 glycoprotein immunogens with endogenous primate CD4 molecules [35]. A mechanism for this important observation is provided in this current study as we clearly demonstrate that mutagenic stabilization of g120, in a similar manner to that achieved by CD4 binding, locks the co-receptor-binding-site into a single conformation that is well recognized by the naïve B cell repertoire in rabbits. This principle might be applicable for viruses that undergo receptor-induced conformational changes to accomplish entry, and for which a vaccine is lacking (e.g., Ebola). Deletion of immunodominant variable regions of Env-based anti-viral subunit vaccines may also have broader applicability.

The protein redesign described here to improve expression revealed some interesting observations relative to recognition by the 17b antibody and implications on the bridging sheet in the coreV3S context. Our earlier data indicated that the previously crystallized core protein could not bind 17b unless induced by CD4 [16]. In this study, the previously described core protein was modified at the base of the V3 loop and at the base of the V1V2 loop. Somewhat unexpectedly, we observed that the newly designed coreV3S protein was recognized by 17b with very high affinity even in the absence of CD4, and that CD4 binding affinity of this protein was markedly improved. A plausible explanation of this modified antigenicity is that restoration of the β12 and β13 strands on the outer domain indirectly aids in formation of bridging sheet elements that are critical for 17b recognition. Restoration of these strands may also impart stability at the base of the Phe 43 cavity, located above (CD4 binding site, see Figure 1). These implications are somewhat in conflict with the unliganded SIV core structure, which shows the bridging sheet β-strands in a non-CD4-bound orientation, but perhaps represent differences in the structural elements present between core and coreV3S proteins and/or differences between HIV and SIV [23]. However, these data are consistent with the initial analysis of 17b recognition, which revealed that 17b binds well to full-length gp120 possessing the V3 loop, but not at all to a V3-loop deleted protein [6], confirmed by our recent studies [28],[35]. Complete V3 loop deletion was performed in the original HIV-1 and SIV crystallized cores proteins. However, because interaction of CD4 with V3 loop-deleted gp120 completely restores 17b binding, this suggests that CD4 can compensate for the (artificial) instability of the bridging sheet region imparted by full truncation of the V3 stem. Yet unresolved, then, is the structure of the receptor-binding sites in the context of the static functional spike (i.e., pre-receptor bound state). The data described above are consistent with the model that the co-receptor-binding site can exist in the context of the static viral spike, but accessibility to antibody is limited unless steric constraints are reduced by receptor engagement.

In previous studies, we have shown that the Phe 43 cavity-filling mutations partially lock gp120 into the receptor/co-receptor-bound conformation [24],[28]. To achieve greater stabilization, we added two pairs of cysteines and additional cavity-filling mutations to core gp120 [24] and analyzed the thermodynamic effects of these mutations on core gp120. Interestingly, we showed clearly that 17b itself can stabilize gp120 into the conformation recognized by CD4 (*B1* reactions) and that the Ds mutations used in this study have no further effect on this conformation (*B2* reactions). However, it is noteworthy that, a relatively constant amount of entropy change (15.1 to 17.3 kcal/mol) was always detected upon addition of CD4 to gp120-17b complexes irrespective of the presence of the stabilizing mutations. We assume that this entropy is either accounted for stabilization of elements distal from the cysteine pairs themselves or, alternatively, results from some unanticipated solvent displacement effects.

Increases in 17b affinity or increased stabilization of the 17b epitope alone were not always associated with all differences in the immunogenicity described here. For example, between 2CC and 3CC, increased 17b affinity correlated very significantly with increased elicitation of CD4i antibodies, although the degree of stabilization achieved was similar in these immunogens. In contrast, the increase in 17b affinity was minimal from 3CC to 4CC, although there was a substantial difference in epitope stabilization. This difference correlated with the enhanced elicitation of CD4i antibodies by 4CC. Therefore, ligand affinity and epitope stabilization both contributed to the overall altered immune responses elicited by the current set of immunogens.

The same set of mutations that stabilized the co-receptor-binding site also stabilized the CD4bs, generally considered a more desired target of the study design because of the neutralization capacity of both CD4 itself and of the CD4bs antibody, b12. In fact, we clearly demonstrate the elicitation of CD4bs antibodies both by ligand cross-competition and by selective adsorption. However, the unmodified cores appear to elicit this type of activity more efficiently than the stabilized cores (see Figure 6A, Figure S6 and Figure S7). It might be that the stabilization process itself subtly altered the CD4-binding surface on gp120 and actually reduced cross-reactivity with natural sequences found on the virus. Alternatively, the stabilized CD4i site perhaps became immuno-dominant and out-competed CD4bs-directed responses. Furthermore, although stabilization of the CD4bs, relative to its starting entropy, approached that of the co-receptor-binding site, the absolute values of CD4-related entropy and affinity did not. Analysis of the sera from one representative animal immunized with coreV3S group compared to one representative animal immunized with the 4CC protein demonstrated a shift of antibody response towards the 17b epitope, correlating with the increased 17b affinity. Consistent with these results, immunization studies using synthetic peptide immunogens indicate that the kinetics of antigen recognition influence epitope-driven repertoire selection and antibody maturation [22]. Achieving slower ligand off-rate may have the potential to improve immune response, although that property may not always be approachable by structure-based design and might be dependent upon the context. This might be in part due to the uncertainty of the factors that define which elements on the surface of a complex protein are most immunogenic. It was suggested that all accessible domains on the surface of a multi-determinant antigen can potentially induce primary B cell responses [36]. However, only those that interact with naive B cells with high affinity will generate an avid antibody response [37].

Here, we demonstrate in this complex model system that it is conformational fixation, associated with increased 17b on-rate and overall affinity, which drives the elicitation of functionally cross-neutralizing antibodies directed toward the gp120 co-receptor binding region. This class of antibodies was extensively studied for their unique properties of posttranslational tyrosine sulfation and preferential VH gene usage [38],[39]. Although to date there are no identified co-receptor binding-site-directed monoclonal antibodies that potently neutralize diverse primary isolates, our recent study implicates antibodies with specificities to this site contribute to neutralization in broadly reactive HIV-1 patient sera [32]. CD4-induced antibodies were also associated with partial control of SHIV challenge in macaques [40]. In the current study, the stabilized immunogens elicited moderate neutralization responses against a few Tier 1 HIV-1 isolates that are typically sensitive to antibodies directed to the gp120 variable loop 3, a component absent in the immunogens tested here. Additional analysis may be warranted to determine if the neutralization activity observed in selected sera is indeed mediated by CD4-induced antibodies as was determined here for the Tier 1 isolates, MW965 and SS1196. Thus, the co-receptor-binding region, usually occluded on most primary isolates, remains an intriguing target due to its conservation, especially if there exists an as yet-to-be-defined subset of antibodies that can access elements of this region on circulating isolates. In addition to CD4-induced responses, the principle established in this study may have important implications for proper stabilization of the CD4bs to generate more broadly cross-reactive and neutralizing antibodies to this heavily shielded, receptor-binding region towards the development of a broadly protective HIV-1 vaccine. Beyond HIV-specific vaccine development, the viral envelope glycoprotein and its ligands under study here provide a model system to establish “proof-of-principle” regarding targeted immunogenicity. Such principles may extend to the design of vaccines against other pathogens capable of humoral immune evasion.

## Materials and Methods

### Molecular Modeling

To design a more optimal V3 truncation, hydrogen bonding at the V3 base was examined in the gp120 core with V3 structure (PDB ID 2B4C). To preserve observed hydrogen bonds, additional residues between the last residue in the β12-strand and the first in the β13-strand were retained. To design a shortened V1/V2 truncation, type II turns were modeled onto strands β2 and β3 to determine the shortest that preserved full β2-hydrogen bonding to strand β21. Lastly, to determine where stabilizing disulfide bonds might be introduced into the gp120 core structure, a distance matrix between all C·β atoms was calculated [41]). All Cβ inter-domain pairs with distances between 3–6 Å were analyzed with explicit modeling disulfide pairs, using the interactive software provided by the program “O” [42].

### Protein Production

Plasmids for the expression of HXBc2 gp120 core and Ds12F123 proteins have been described before [43]. Plasmids expressing other immunogens (listed in Figure 1B) were derivated either by Quick change PCR mutagenesis (for 3CC and 4CC) or by de novo gene synthesis (coreV3S and 2CC). All immunogen proteins were expressed in serum-free medium by transient transfection of HEK293T cells and purified over antibody columns as described earlier [28]. The core protein was purified over b12 affinity column and all proteins with V3S modifications were purified over 17b affinity columns as was the Ds12F123 protein. Expression and purification of proteins used in serum adsorption analyses have been described elsewhere [31],[32].

### ELISA

The antigenicity of WT and mutant envelope proteins (Figure 2) and the anti-gp120 antibody titers in immunized sera were determined by ELISA analyses as described in Dey et el., 2007 [28].

### Isothermal Titration Calorimetry (ITC)

All ITC reactions were performed at 37°C as described in Dey et al., 2007 [28]. The concentration of gp120 in the sample cell was approximately 4 µM and that of sCD4 or 17b in the syringe was approximately 40 µM. The molar concentrations of the proteins were calculated using the following molar extinction coefficients: core, 1.35; Ds12F123, 1.4; coreV3S, 1.33; 2CC, 1.5; 3CC, 1.5; 4CC, 1.5; sCD4, 0.93 and IgG17b, 1.47. The specific activity of sCD4 and 17b was determined as described previously [28],[44]. The values for enthalpy (Δ*H*), entropy (Δ*S*), and the association rate constant (*K_a_*) were obtained by fitting the data to a nonlinear least-squares analysis with Origin software. For the second step of a two-step reaction, gp120 concentration was recalculated as per the final volume at the end of the first reaction, which is equal to the sample cell volume plus the total volume of the first injectant. Before the second titration, the sample volume equivalent to the volume of the first injectant was removed from the sample cell.

### SPR Kinetic Binding Analysis

All kinetic reactions were performed at RT on a Biacore3000 surface plasmon resonance spectrometer. To prepare binding surfaces with approximately 500 RU per cell, ligands (7 µg/ml in 10 mM NaOAc, pH 5.5 buffer) were immobilized on CM5 chip by the amine coupling method following manufacturer's protocol. The reference cell received only NaOAc buffer. Analytes were serially diluted in the HEPES-EP reaction buffer at concentrations ranging from 6.2 nM to 400 nM for sCD4 and b12 or 6.2 nM to 50 nM for 17b. Association was allowed for 3 min at 30 µl/min. To determine 17b binding in the presence of CD4, gp120 dilutions were pre-incubated for 30 min at RT with 10-fold molar excess of 2-domain sCD4. Dissociation was determined by washing off bound analyte over the next 5 min. The chip surface was regenerated with two injections (60 sec each) of 10 mM Glycine, pH 3.0. The kinetic rate constants were obtained by fitting the curves to 1∶1 Langmuir binding model using BIAevaluation software.

### Animal Immunization

Approximately 12 weeks old female New Zealand White rabbits were inoculated with 50 µg of affinity-purified protein formulated in GlaxoSmithKline Adjuvant System AS01B, injected intramuscularly by splitting the protein-adjuvant mix in the two hind legs at 4 weeks intervals. Serum was collected 8–10 days after each inoculation. Serum preparation and heat inactivation of complement systems were performed as described earlier [28]. All rabbits were housed and maintained in the AAALAC-accredited BIOCON, Inc (Rockville, MD) under specific pathogen-free conditions. All experiments were approved by the Animal Care and Use Committee of the Vaccine Research Center and BIOCON, Inc.

### Virus Neutralization Assays

Production and neutralization of pseudotyped HIV-1isolates were described earlier [10],[28],[31]. To control for non-specific effects in the assay, preimmune sera, BSA-AS01B-immunized antisera and a pseudovirus expressing murine leukemia virus envelope were analyzed [28],[45]. Neutralization of HIV-2 strain 7312A/V434M was performed in the presence of non-neutralizing levels of sCD4 and analyzed as previously described [28],[29].

### Serum Adsorptions

Antibody populations directed towards the CD4bs or the CD4i sites were separated by absorbing rabbit immune sera on protein-coated dynabeads as previously described [31]. In brief, sera were diluted between 1∶4 and 1∶20 in DMEM/10% FBS and 1000 µl of diluted sera was incubated with 500 µl of beads at room temperature for 30 minutes, followed by a second adsorption with 250 ul of beads. After serum adsorption, beads were removed with a magnet followed by centrifugation and were stored in PBS/0.2%BSA/0.02% sodium azide buffer at 4°C.

## Supporting Information

Figure S1 Reducing SDS-PAGE of unmodified and stabilized core glycoproteins. All glycoproteins shown except the original core were purified by 17b affinity chromatography. The core was purified by b12 affinity chromatography because it is poorly recognized by 17b. The 17b antibody selects for a hyperglycosylated form of the modified core variants, in part accounting for the slightly slower migration of the 17b-purified glycoproteins in the gel.
(2.33 MB TIF)

Figure S2 Binding titers of sera from rabbits immunized with envelope variants as determined by ELISA. Affinity purified coreV3S protein (2 µg/ml) was coated on ELISA plates, reacted with fivefold serial dilutions of different immune sera and detected with anti-rabbit IgG-peroxidase conjugated secondary antibody. Arrows indicate end point titers, defined as the last reciprocal serum dilution at which the optical density signal was greater than twofold over the signal detected with the preimmune sera. A. Comparison of titers following two, three and four inoculations of coreV3S protein. B. Comparison of titers among different groups of immune rabbit sera following four inoculations.
(1.82 MB TIF)

Figure S3 Neutralization profile of fivefold diluted rabbit immune sera tested against a panel of HIV-1 and HIV-2 isolates. All sera tested were collected after four inoculations. Neutralization by preimmune sera was used as negative control for serum reactivity.
(1.84 MB TIF)

Figure S4 Neutralization ID_50_ titers of selected HIV-1 isolates by Env-immunized rabbit anti-sera.
(1.65 MB TIF)

Figure S5 Elicitation of CD4i antibodies in guinea pigs following immunization with stabilized core variants. A. Schematic representation of immunogens used. B. IC_50_ titers of HIV-2 neutralization by guinea pig sera collected after 4 inoculations.
(1.90 MB TIF)

Figure S6 ELISA analysis comparing inhibition of sCD4 binding to envelope glycoprotein by various groups of rabbit immune sera (im.s.) collected after four inoculations. A. Validation of the sCD4-inhibition assay. ELISA plates coated with core gp120 (2 µg/ml) protein were preincubated with fivefold dilutions of rabbit immune sera or ligands, reacted with 0.8 µg/ml of sCD4 followed by biotinylated guinea pig IgG anti-CD4, and detected with HRP-conjugated streptavidin. BSA-immunized rabbit serum and WTgp120-immunized rabbit serum were used as negative and positive control respectively for serum interactions. Unlabeled IgGb12 and IgG17b were used as controls for ligand binding. Binding of sCD4 in presence of the lowest concentration of BSA-immunized serum was considered 100%. Margins of error from duplicate wells were negligible. A. Validation of the sCD4-inhibition assay. B. Range of residual sCD4 binding to coreV3S protein in the presence of the highest concentration (20-fold dilution) of various immune sera. Values obtained were normalized against 100% binding in the presence of 2500-fold diluted BSA-immunized sera. The horizontal lines indicate mean values with standard errors of mean (SEM) for each group of sera. C. Same as in B except blocking of CD4 binding by each group of sera was detected with the corresponding protein immunogen coated on ELISA plate.
(1.51 MB TIF)

Figure S7 ELISA analysis comparing inhibition of b12 binding to coreV3S protein by various groups of rabbit immune sera (im.s.). ELISA plates, coated with 2 µg/ml of protein, were preincubated with fivefold dilution of rabbit immune sera or ligands for 45 min at RT, reacted with biotinylated b12 (0.056 µg/ml of final concentration; M. Roederer, Conjugation of monoclonal antibodies, August 2004; http://www.drmr.com/abcon) for 30 min at RT and detected with 1∶250 dilution of HRP-conjugated streptavidin. BSA-immunized rabbit serum was used as negative control and either full-length gp120 (WTgp120)-immunized rabbit serum (panel A; Dey et al., 2007) or coreV3S-immunized rabbit serum (animal ID# 4; Panel B) were used as positive controls for serum interactions. Unlabeled IgGb12 and IgG17b were used as positive and negative controls respectively for ligand binding. Binding of b12 in the presence of the lowest concentration (2500-fold dilution) of BSA-immunized serum was considered 100%. Margins of error from duplicate wells were negligible. A. Validation of the b12-inhibition assay. B. Inhibition of b12 binding to coreV3S protein by immune sera tested over a range of dilution. C. Relative b12 binding to coreV3S protein in the presence of the highest concentration (20-fold dilution) of various immune sera. The dotted horizontal line indicates 100% b12 binding in the presence of 2500-fold diluted BSA-immunized sera. The mean values of b12 binding with standard errors of mean (SEM) for each group of sera are shown. Seven sera from coreV3S group and nine sera from each of 2CC, 3CC and 4CC groups were tested.
(2.39 MB TIF)
