# Supplementary figures and images for: Structure-Based Stabilization of HIV-1 gp120 Enhances Humoral Immune Responses to the Induced Co-Receptor Binding Site
Source: PLoS Pathog. 2009 May 29;5(5):e1000445. doi: 10.1371/journal.ppat.1000445 (PMC2680979; doi:10.1371/journal.ppat.1000445)

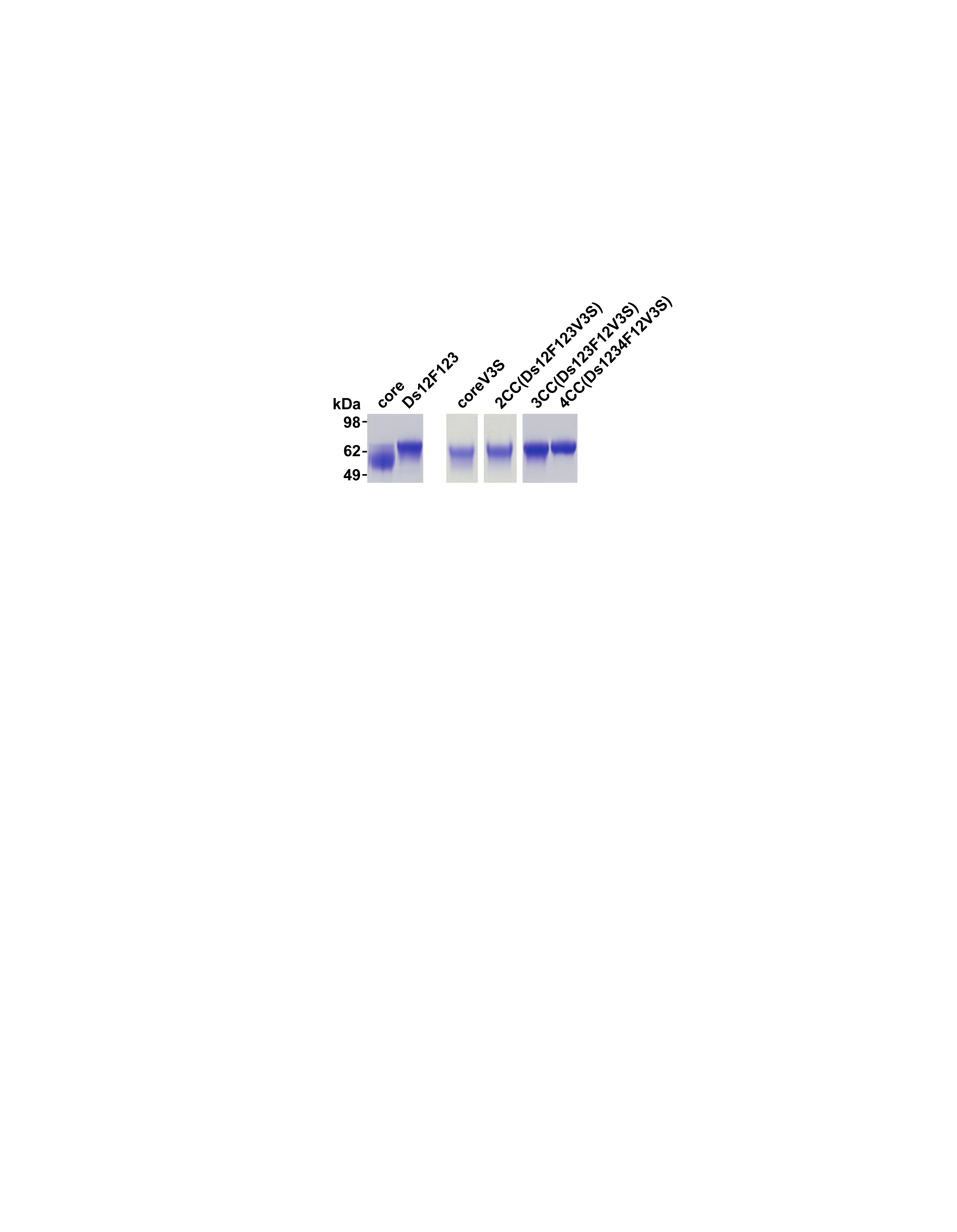

Supplement: Figure S1 — Reducing SDS-PAGE of unmodified and stabilized core glycoproteins. All glycoproteins shown except the original core were purified by 17b affinity chromatography. The core was purified by b12 affinity chromatography because it is poorly recognized by 17b. The 17b antibody selects for a hyperglycosylated form of the modified core variants, in part accounting for the slightly slower migration of the 17b-purified glycoproteins in the gel. (2.33 MB TIF) [file ppat.1000445.s001.tif]

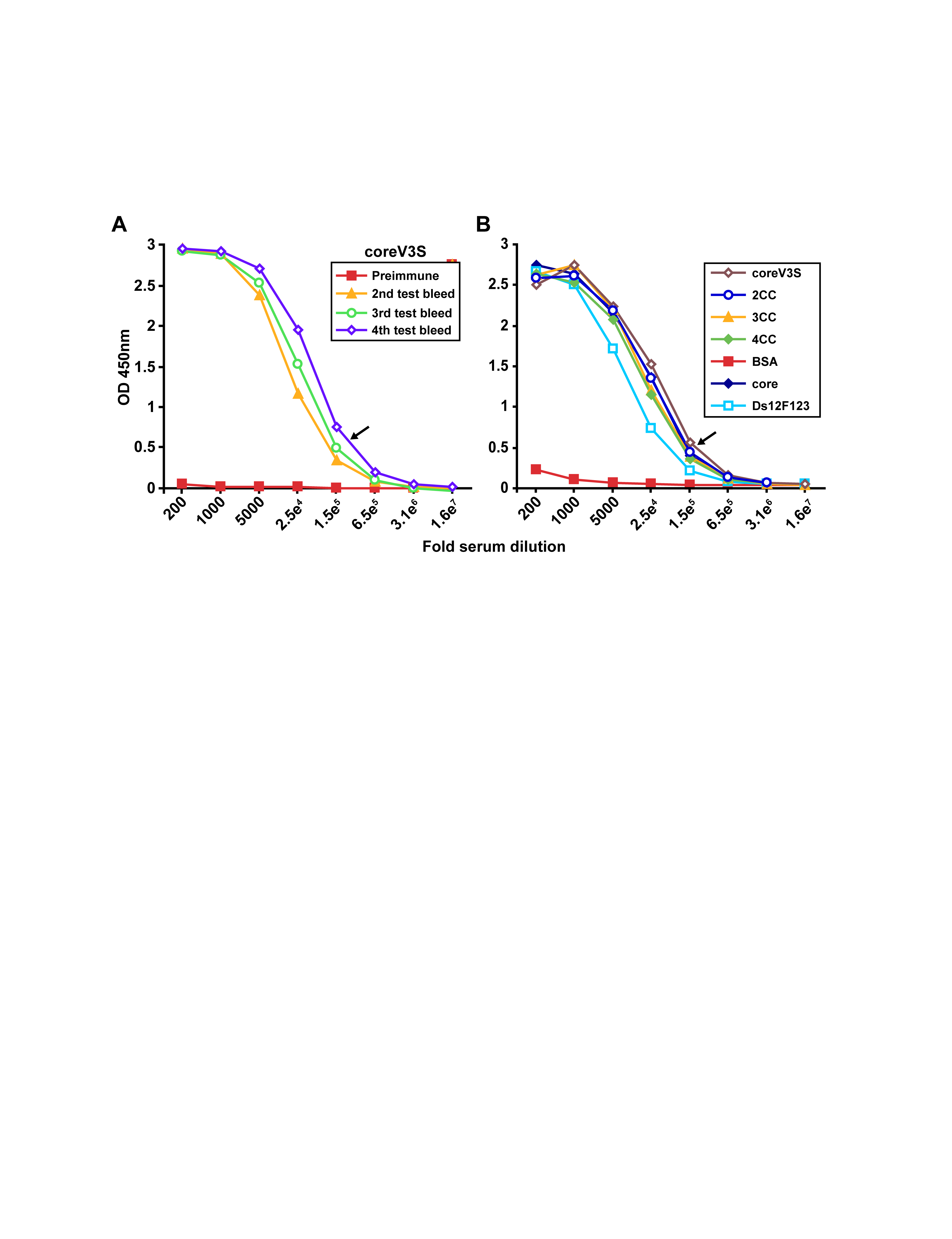

Supplement: Figure S2 — Binding titers of sera from rabbits immunized with envelope variants as determined by ELISA. Affinity purified coreV3S protein (2 µg/ml) was coated on ELISA plates, reacted with fivefold serial dilutions of different immune sera and detected with anti-rabbit IgG-peroxidase conjugated secondary antibody. Arrows indicate end point titers, defined as the last reciprocal serum dilution at which the optical density signal was greater than twofold over the signal detected with the preimmune sera. A. Comparison of titers following two, three and four inoculations of coreV3S protein. B. Comparison of titers among different groups of immune rabbit sera following four inoculations. (1.82 MB TIF) [file ppat.1000445.s002.tif]

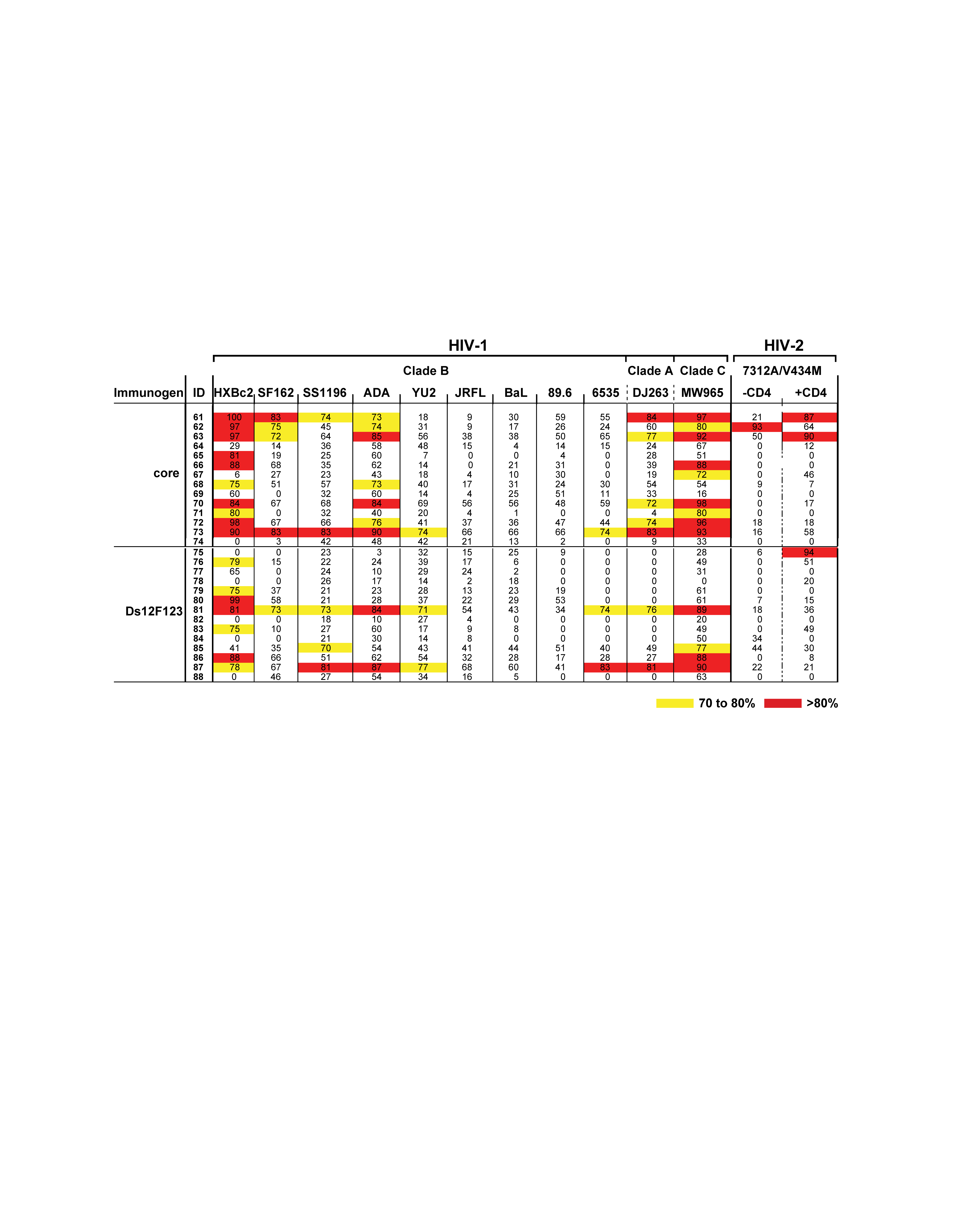

Supplement: Figure S3 — Neutralization profile of fivefold diluted rabbit immune sera tested against a panel of HIV-1 and HIV-2 isolates. All sera tested were collected after four inoculations. Neutralization by preimmune sera was used as negative control for serum reactivity. (1.84 MB TIF) [file ppat.1000445.s003.tif]

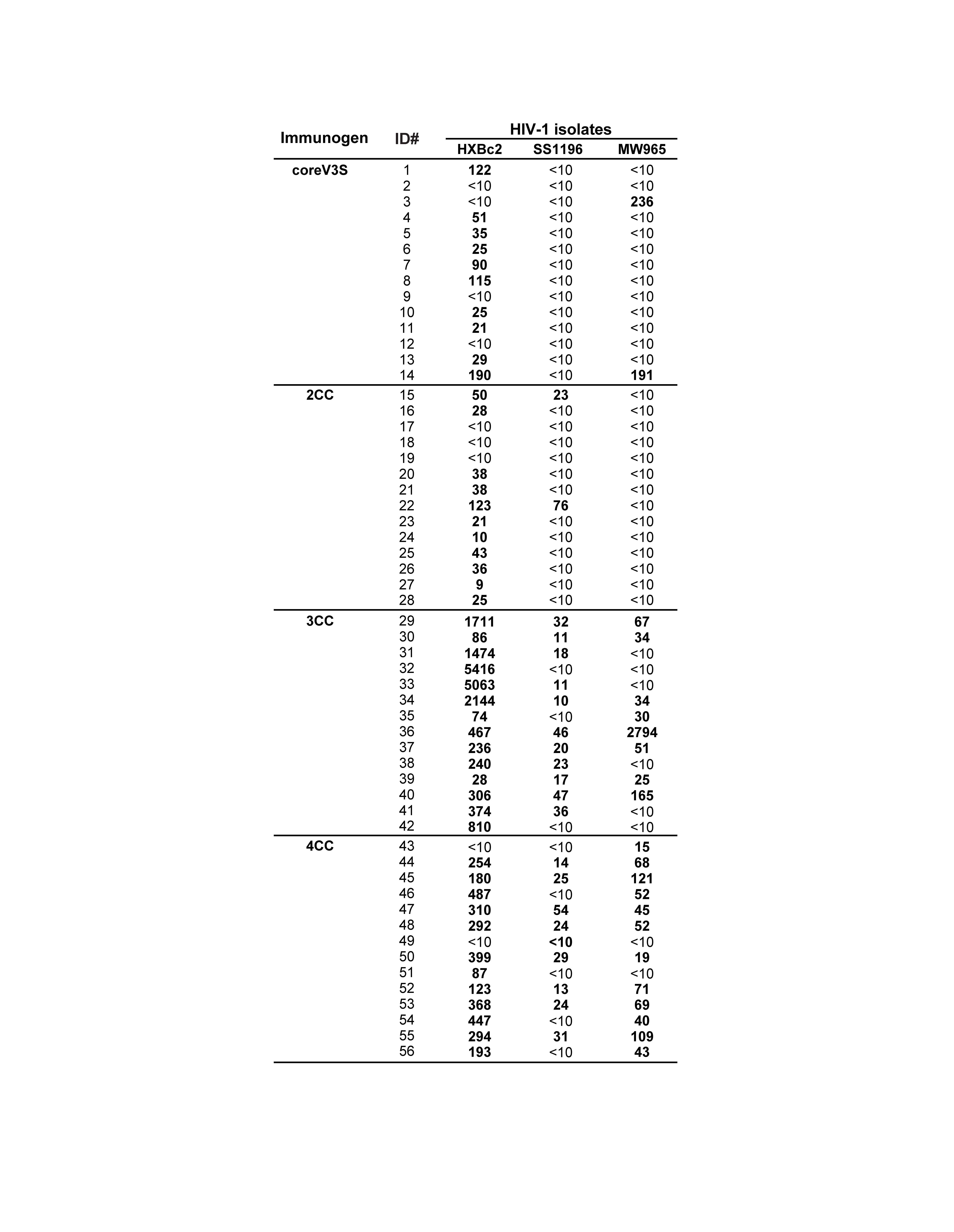

Supplement: Figure S4 — Neutralization ID50 titers of selected HIV-1 isolates by Env-immunized rabbit anti-sera. (1.65 MB TIF) [file ppat.1000445.s004.tif]

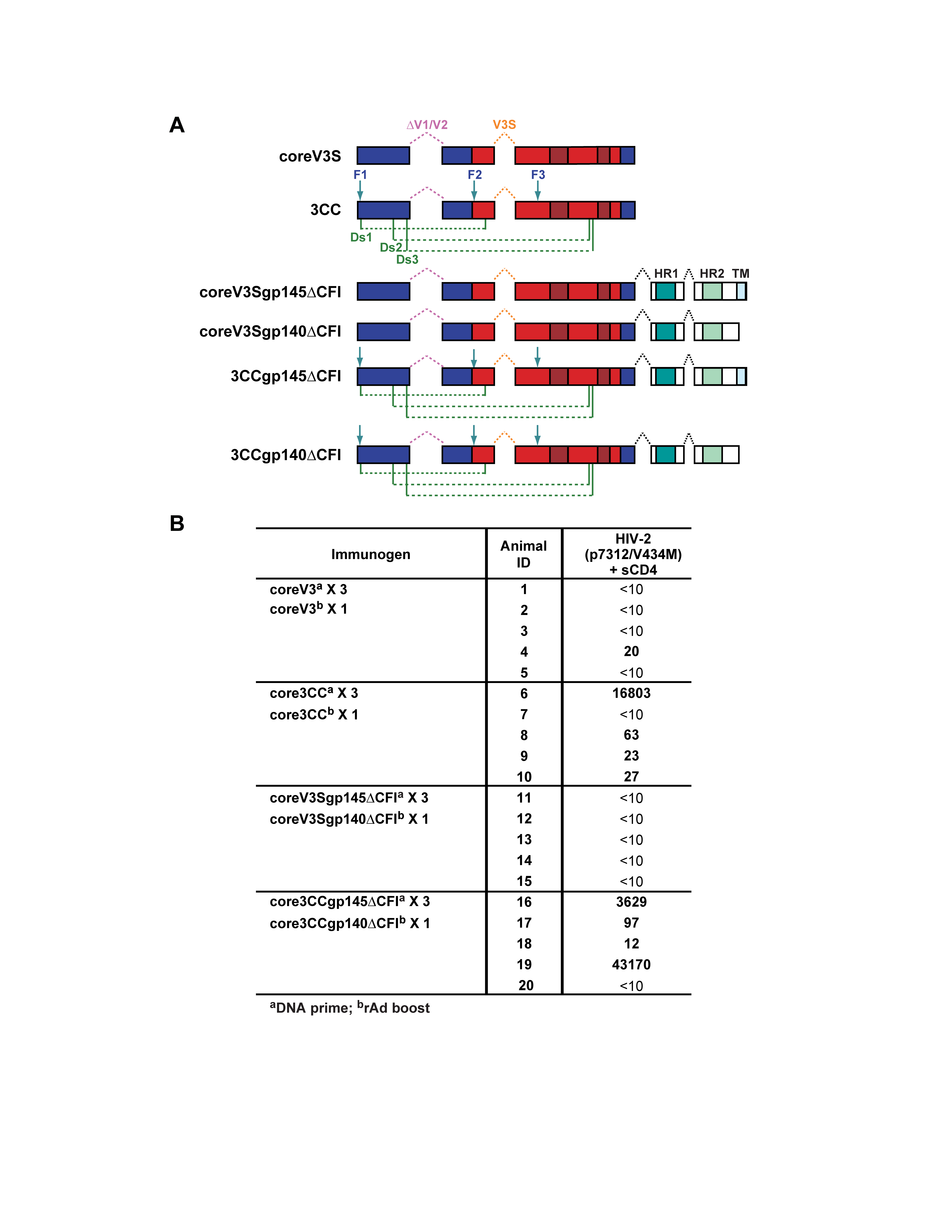

Supplement: Figure S5 — Elicitation of CD4i antibodies in guinea pigs following immunization with stabilized core variants. A. Schematic representation of immunogens used. B. IC50 titers of HIV-2 neutralization by guinea pig sera collected after 4 inoculations. (1.90 MB TIF) [file ppat.1000445.s005.tif]

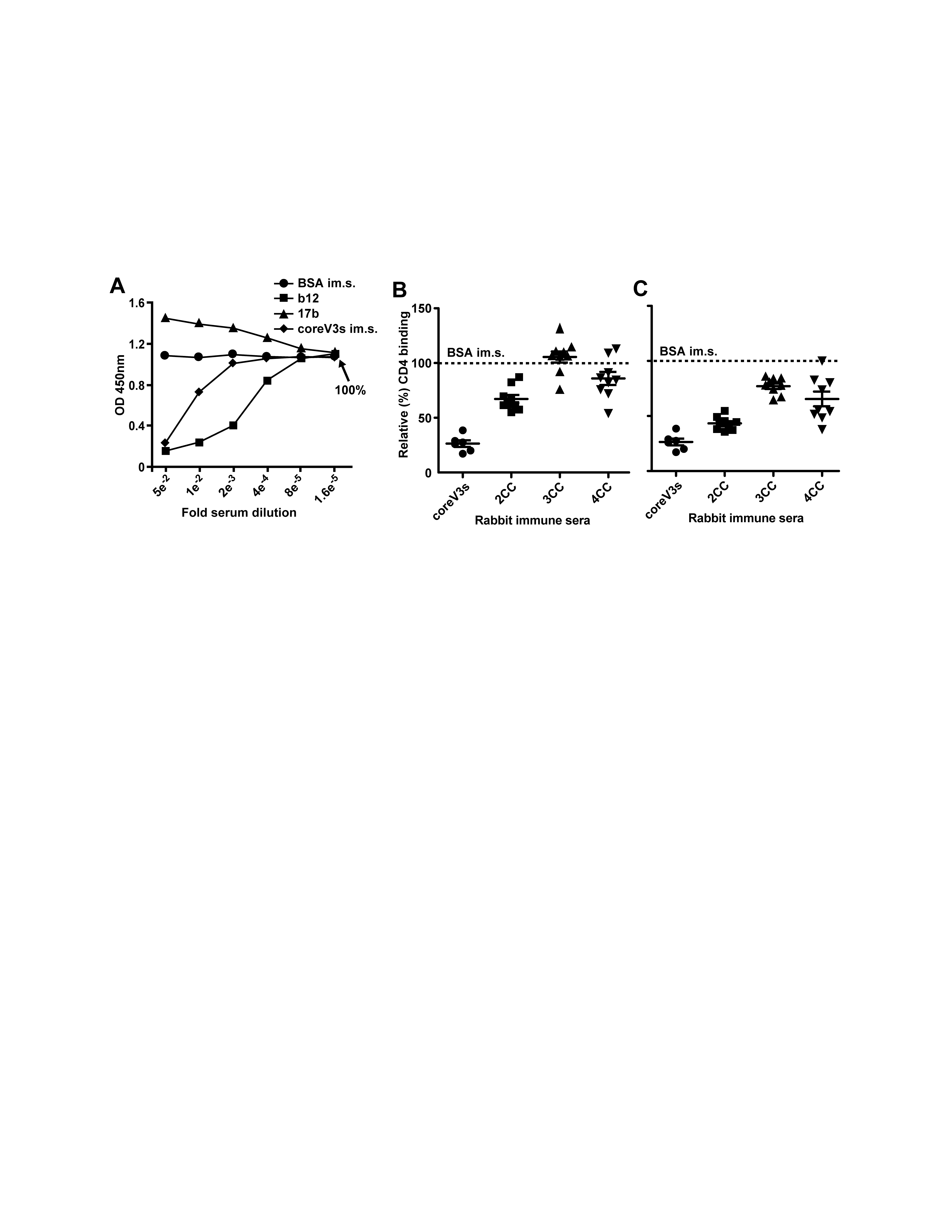

Supplement: Figure S6 — ELISA analysis comparing inhibition of sCD4 binding to envelope glycoprotein by various groups of rabbit immune sera (im.s.) collected after four inoculations. A. Validation of the sCD4-inhibition assay. ELISA plates coated with core gp120 (2 µg/ml) protein were preincubated with fivefold dilutions of rabbit immune sera or ligands, reacted with 0.8 µg/ml of sCD4 followed by biotinylated guinea pig IgG anti-CD4, and detected with HRP-conjugated streptavidin. BSA-immunized rabbit serum and WTgp120-immunized rabbit serum were used as negative and positive control respectively for serum interactions. Unlabeled IgGb12 and IgG17b were used as controls for ligand binding. Binding of sCD4 in presence of the lowest concentration of BSA-immunized serum was considered 100%. Margins of error from duplicate wells were negligible. A. Validation of the sCD4-inhibition assay. B. Range of residual sCD4 binding to coreV3S protein in the presence of the highest concentration (20-fold dilution) of various immune sera. Values obtained were normalized against 100% binding in the presence of 2500-fold diluted BSA-immunized sera. The horizontal lines indicate mean values with standard errors of mean (SEM) for each group of sera. C. Same as in B except blocking of CD4 binding by each group of sera was detected with the corresponding protein immunogen coated on ELISA plate. (1.51 MB TIF) [file ppat.1000445.s006.tif]

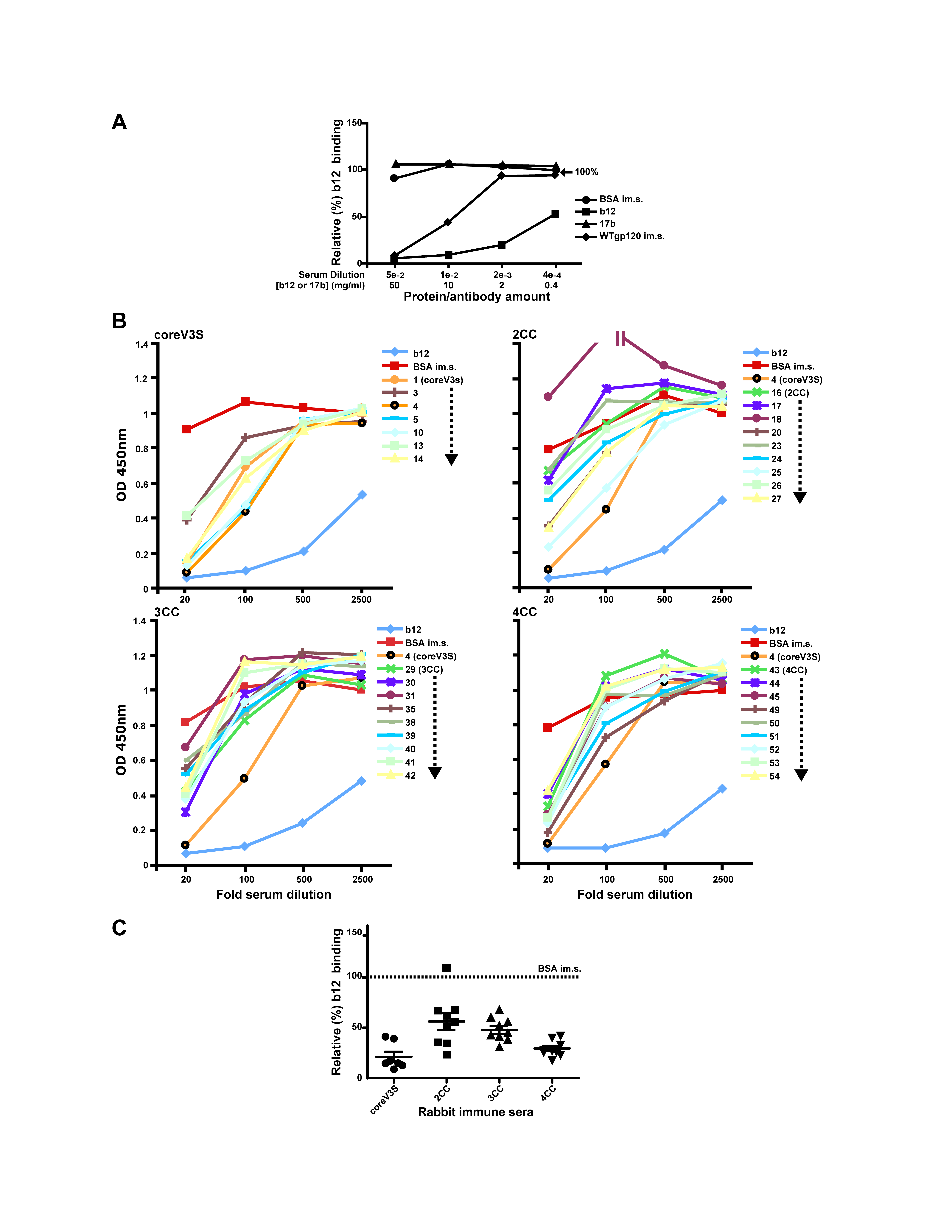

Supplement: Figure S7 — ELISA analysis comparing inhibition of b12 binding to coreV3S protein by various groups of rabbit immune sera (im.s.). ELISA plates, coated with 2 µg/ml of protein, were preincubated with fivefold dilution of rabbit immune sera or ligands for 45 min at RT, reacted with biotinylated b12 (0.056 µg/ml of final concentration; M. Roederer, Conjugation of monoclonal antibodies, August 2004; http://www.drmr.com/abcon) for 30 min at RT and detected with 1∶250 dilution of HRP-conjugated streptavidin. BSA-immunized rabbit serum was used as negative control and either full-length gp120 (WTgp120)-immunized rabbit serum (panel A; Dey et al., 2007) or coreV3S-immunized rabbit serum (animal ID# 4; Panel B) were used as positive controls for serum interactions. Unlabeled IgGb12 and IgG17b were used as positive and negative controls respectively for ligand binding. Binding of b12 in the presence of the lowest concentration (2500-fold dilution) of BSA-immunized serum was considered 100%. Margins of error from duplicate wells were negligible. A. Validation of the b12-inhibition assay. B. Inhibition of b12 binding to coreV3S protein by immune sera tested over a range of dilution. C. Relative b12 binding to coreV3S protein in the presence of the highest concentration (20-fold dilution) of various immune sera. The dotted horizontal line indicates 100% b12 binding in the presence of 2500-fold diluted BSA-immunized sera. The mean values of b12 binding with standard errors of mean (SEM) for each group of sera are shown. Seven sera from coreV3S group and nine sera from each of 2CC, 3CC and 4CC groups were tested. (2.39 MB TIF) [file ppat.1000445.s007.tif]
